# INTERNET LITERACY FROM GRANDCHILDREN OR YOUNGER GENERATION: A NEW DIRECTION FOR GERONTECHNOLOGY

**DOI:** 10.1093/geroni/igac059.2193

**Published:** 2022-12-20

**Authors:** Ananya Sarker, Jeffrey Kahana, Eva Kahana

**Affiliations:** Case Western Reserve University, Cleveland, Ohio, United States; Mount Saint Mary College, Newburgh, New York, United States; Case Western Reserve University, Cleveland, Ohio, United States

## Abstract

Internet literacy offers a modern set of tools that can help older adults achieve the three pillars of successful aging, especially in the post-pandemic world. In the United States, older adults have shown significant growth in the usage of the internet and online services. However, information and social connectivity for older adults with lower incomes and education levels still lag. The current pandemic raises the public question of whether internet access and education should be deemed as a basic human right. Internet access is even more critical for older adults who need additional technology access and training, tailored to fit their varying experience, disability, and cognitive ability levels. The pandemic has demanded numerous technological skills for daily life: videoconferencing to carry on work, ordering groceries, taking exercise classes online, talking to family members and healthcare providers, and now registering for vaccination. Thus older adults need help with technology use more than ever. We need awareness of the “digital divide” and collaboration from designers, developers, services, products, and programs or interventions from various public and private sources. Identifying social and logistical barriers to technology literacy, we propose a conceptual model of using intergenerational training strategies to ameliorate the digital divide experienced by older adults. In this paper, we will review successful intergenerational programs like Students to Seniors, and Zoomers to Boomers, and introduce a similar model for internet literacy. The intergenerational mentoring model is advocated due to its wide-ranging potential benefits for both the older generation and the younger generation.

